# CHAF1B: the hidden culprit behind sorafenib resistance in hepatocellular carcinoma

**DOI:** 10.7150/ijms.118471

**Published:** 2025-09-22

**Authors:** Wenfeng Zhuo, Jiali Zhao, Zhouying Zheng, Shanglin Cai, Guifang Zeng, En Lin, Zirui Bai, Bo Wang, Yingbin Jia, Peiping Li, Jian Li

**Affiliations:** 1 Department of Hepatobiliary Surgery, the Fifth Affiliated Hospital, Sun Yat-sen University 519000 Zhuhai, Guangdong, China.; 2 Department of Urology Surgery, the Fifth Affiliated Hospital, Sun Yat-sen University, Zhuhai 519000, Guangdong, China.

**Keywords:** hepatocellular carcinoma, sorafenib resistance, PI3K/Akt/HIF-1α signaling pathway, CHAF1B

## Abstract

**Introduction:** Chromatin assembly factor 1B (CHAF1B), a pivotal regulator of chromatin assembly following DNA replication, has been implicated in oncogenic processes. However, its role in sorafenib resistance and potential anti-tumor mechanisms in hepatocellular carcinoma (HCC) remain unclear. This study has sought to elucidate CHAF1B's therapeutic potential and its potential synergistic role with sorafenib in overcoming chemoresistance.

**Methods:** In this study, bioinformatics, immunohistochemistry, western blot, CCK8, colony formation, transwell migration and invasion, and flow cytometry were performed to analyze the correlation between CHAF1B and sorafenib resistance in HCC. Furthermore, RNA sequencing (RNA-seq), combined with signaling pathway-specific inhibitors, was used to elucidate the specific role of CHAF1B in sorafenib resistance of HCC and its related mechanism.

**Results:** CHAF1B was significantly upregulated in HCC tissues and sorafenib-resistant HCC cells, with elevated expression correlated with reduced survival probability in HCC patients. Moreover, high CHAF1B levels predicted poorer clinical outcomes in sorafenib-treated patients. Functional assays revealed that CHAF1B promotes HCC cell proliferation, migration, and invasion, while also enhancing resistance to sorafenib. In contrast, knockdown of CHAF1B significantly increased sorafenib-induced inhibition of proliferation and cell death. Mechanistically, CHAF1B facilitated malignant phenotypes via activation of the PI3K/Akt/HIF-1α pathway. Furthermore, blockade of PI3K/Akt/HIF-1α signaling partially attenuated the CHAF1B-mediated sorafenib resistance.

**Conclusion:** CHAF1B is a key regulator of sorafenib resistance, and targeting CHAF1B in conjunction with sorafenib may represent a promising therapeutic approach for HCC by modulating the PI3K/Akt/HIF-1α signaling axis.

## Introduction

Hepatocellular carcinoma (HCC) is the sixth most common cancer and the third leading cause of cancer-related deaths globally 1. Due to the challenges of early diagnosis, most patients with HCC are diagnosed at advanced stages, making systemic therapy the most effective treatment option for them 2, 3. Sorafenib, a multi-tyrosine kinase inhibitor (mTKI) primarily targeting the RAF/MEK/ERK signaling pathway, was the first drug approved by the U.S. Food and Drug Administration (FDA) for first-line systemic treatment of advanced HCC 4, 5. Despite continuous efforts to develop clinically effective strategies for targeting HCC, sorafenib has remained the globally recognized first-line treatment for the disease 6, 7. However, the efficacy and effectiveness of sorafenib treatment are frequently limited by intrinsic or acquired resistance to the drug 8, 9. Therefore, it is necessary to investigate the mechanisms underlying sorafenib resistance and explore therapeutic strategies to enhance drug efficacy in HCC.

Chromatin Assembly Factor-1 (CAF-1) is a heterotrimeric histone chaperone composed of p48, p60, and p150 proteins, which plays significant roles in regulating chromatin assembly in conjunction with DNA replication and repair 10. Chromatin assembly factor 1B (CHAF1B) is the p60 subunit of the CAF-1 complex, which is located in the nucleolus during interphase and at the site of DNA replication in the S phase, and is positively associated with cell proliferation 11. Recent evidence increasingly indicates that dysregulation of CHAF1B expression is linked to malignant phenotype and poor prognosis in various tumors. To be specific, elevated levels of CHAF1B have been observed in breast, oral, prostate, and salivary gland carcinomas, as well as in skin melanoma. Furthermore, its expression is closely linked to tumor metastasis, invasion, and adverse clinical outcomes 12-17. In addition, in the hematopoietic system, CHAF1B is essential for normal hematopoiesis, while its overexpression promotes leukemia 18. Notably, CHAF1B has been reported to protect against cisplatin-induced cytotoxicity in lung adenocarcinoma and to induce radioresistance by promoting DNA damage repair in nasopharyngeal carcinoma 19, 20. Recently, CHAF1B was reported to play an important role in the regulation of proliferation and cell apoptosis in HCC 21. Moreover, our preliminary bioinformatic analysis suggested that the expression of CHAF1B is positively correlated with overall survival (OS) in HCC patients treated with sorafenib, indicating that CHAF1B may play a role in sorafenib resistance in HCC. However, the mechanism underlying CHAF1B-mediated sorafenib resistance remains unclear.

In this study, we demonstrated that CHAF1B was upregulated in HCC, and high expression levels of CHAF1B contributed to sorafenib resistance in HCC. Knockdown of CHAF1B enhanced sorafenib-induced proliferation inhibition and cell death by reducing PI3K/Akt/HIF-1α activation.

## Materials and Methods

### Cell culture

The human HCC cell lines HepG2, Huh7 were purchased from Procell Life Science & Technology Co., Ltd. (Cat. No. CL-0103, CL-0120, China) in 2023. Both the cells passed the short tandem repeat (STR) analysis and mycoplasma contamination detection. All the cell lines were maintained in Dulbecco's Modified Eagle Medium (DMEM; Gibco, USA), enriched with 10% fetal bovine serum (FBS; Gibco, USA), and supplemented with 100 U/mL of penicillin and 100 U/mL of streptomycin (both from Sigma-Aldrich, USA). Cultures were incubated at 37 °C in a controlled environment with 5% CO_2_ and 21% O_2_.

### Reagents and antibodies

The information for reagents and antibodies used in this study are presented in Table S1.

### Patients and specimens

Liver tissues samples were obtained from 99 patients with HCC who underwent surgical resection at the Fifth Affiliated Hospital of Sun Yat-sen University (Zhuhai, China) between January 2010 and March 2021. Criteria for patient inclusion were as follow: (1) Adult patients (≧18 years old); (2) Histopathologically confirmed HCC; (3) An exclusion of patients with other types of malignancy; (4) No prior chemotherapy, immunotherapy, or radiotherapy before surgery; (5) An exclusion of recurrent HCC patients. Fresh-frozen tissue were stored at -80°C for subsequent western blot analysis, while matched formalin-fixed, paraffin-embedded (FFPE) specimens were prepared for immunohistochemical studies. The baseline clinicopathological characteristics of the patients are summarized in Table S2.

This research was approved by the Institutional Ethics Committee of the Fifth Affiliated Hospital of Sun Yat-sen University (NO. 00337), and written informed consent was obtained from all participants.

### Cell transfection

To achieve stable overexpression or knockdown of CHAF1B in human HCC cell lines, Huh7 and HepG2 cells were transfected with the following lentiviral constructs (HanBio, Shanghai, China): CHAF1B overexpression plasmid (CHAF1B) and corresponding empty vector (control); CHAF1B knockdown construct (shCHAF1B) and scrambled shRNA control. For transfection, 2 × 10^5^ cells were seeded in 6-well plates. After 24 h, the cells were transfected with lentivirus and maintained in a humidified incubator at 37 °C. After 48 h, stable transfected cell lines were selected using DMEM supplemented with 10% FBS and 2μg/mL puromycin. Western blot, RT-qPCR and Immunofluorescence analysis were performed to verify the transfection efficiency.

### Cell proliferation assay

3000 cells per well were seeded into 96-well microplates (Corning, USA). At the indicated times, the cells were treated using cell counting kit-8 (CCK8, MedChemExpress, Cat. No. HY-K0301). Then, cells were incubated at 37 °C for 2 h, absorbance was measured using the iMark™ Microplate Absorbance Reader (Bio-Rad, iMark, USA) at 450 nm. All experiments were repeated five times.

### Colony formation assay

1000 cells per well were seeded into 6-well plate. After 24 h, the medium was replaced with fresh medium supplemented with or without 2.5 μM sorafenib. The cells were washed with PBS and fresh medium supplemented with or without 2.5 μM sorafenib was replaced every 3 days. Following a 2-week incubation, the cells were fixed in 4% paraformaldehyde for 20 min and then stained with 0.1% crystal violet for 30 min. The colonies were counted using a transmission light microscope (EVOS FL Auto, Life Technologies, USA). All experiments were repeated five times.

### Cell viability assays

10,000 cells per well were seeded into 96-well microplates (Corning, USA). After 12 h, the cells were treated with various concentrations of sorafenib (0.5, 1, 2, 4, 8, 16, 32, and 64 μM). Following a 48 h incubation, cell viability was assessed using CCK8 assays (MedChemExpress, Cat. No. HY-K0301). The plates were subsequently incubated at 37 °C for an additional 2 h. Absorbance readings were taken at 450 nm with the iMark™ Microplate Absorbance Reader (Bio-Rad, USA). Each experiment was repeated with five independent replicates.

### RNA isolation and quantitative real-time polymerase chain reaction

The RNA isolation and quantitative real-time polymerase chain reaction (RT-qPCR) were performed as previously described^22^, Briefly, total RNA was extracted from the cultured cells using Trizol reagent (Ambion, USA) according to the manufacturer's instructions. The extracted RNA was then reverse-transcribed into cDNA using HiScript®III RT SuperMix (Vazyme, China) for subsequent quantitative real-time PCR (qPCR) detection. qPCR was conducted according to the manufacturer's instructions, using the SYBR qPCR Master Mix kit (Vazyme, China) on a CFX Connect real-time system (Bio-Rad, California, USA). Fold changes of gene expression level were determined by using the relative quantification 2^-△△CT^ method, normalized to β-actin. The sequences of all primers used in are listed in Table 1.

### Transwell migration and invasion assays

For migration assays, 500 μL of medium containing 20% FBS was first added to a 24-well plate. Subsequently, 6 × 10^4^ cells suspended in 200μL serum-free medium were added to the upper transwell chamber (8µm, Corning, USA). For the invasion assays, a combination of Matrigel (Corning, USA) and serum-free medium was applied to the upper chamber of the transwell before seeding the cells. Following a 36 h incubation period in a 5% CO_2_ atmosphere at 37 °C, any cells that remained on the upper surface of the membrane were carefully removed. The migrated cells in the lower compartment were fixed with 4% paraformaldehyde (Biosharp, China) for 30 min and stained with 0.1% crystal violet for 20 min. Finally, the cells were washed with PBS and counted using a transmission light microscope (EVOS FL Auto, Life Technologies, USA). Each experiment was repeated with five independent replicates.

### Cell apoptosis analysis detected by flow cytometric

Flow cytometric analysis was performed following the procedure described previously 23. Briefly, 5 × 10^5^ cells were seeded into a 6-well plate. After 12 h, the medium was replaced with fresh medium supplemented with 5 μM sorafenib. Following a 24 h or 48 h incubation, the cells were collected and washed twice with PBS. Cell apoptosis was assessed using Apoptosis Detection Kit (Annexin V-APC/PI, Procell Life Science & Technology Co., Ltd., Cat. No. P-CA-207). The percentage of Annexin V-APC-positive cells was measured by polychromatic analytical flow cytometry (Beckman, Cytoflex LX, USA).

### Western blot

Western blot (WB) was performed as previously described 24. Briefly, cells or tissue were lysed in sample buffer, homogenized, and denatured with β-mercaptoethanol (Sigma-Aldrich, USA). Protein was separated by SDS-PAGE gel (Epizyme, China) and then transferred onto the 0.45µm PVDF membrane, followed by blocking with 5% BSA for 1.5 h at room temperature. Subsequently, the membrane was incubated with primary antibody overnight at 4℃, and then incubated with horseradish peroxidase (HRP) conjugated secondary antibodies for 1 h at room temperature. In the end, the automatic fluorescence/chemiluminescence image analysis system (Tanon, Shanghai, China) was used to detect and acquire images. Primary antibody dilutions were as follows: β-actin (1:4000), CHAF1B (1:1000), PI3K p85 (1:1000), p-PI3K p85 (1:1000), Akt (1:1000), p-Akt (Ser473) (1:1000), HIF-1α (1:1000), Cleaved Caspase-3 (1:1000). The targeted bands were analyzed by ImageJ software (v1.8.0; National Institutes of Health, USA). β-actin served as the internal control and relative protein expression was normalized to β-actin. Detailed antibody information is provided in Table S1.

### Immunofluorescence

2 × 10^5^ cells were seeded onto climbing slices in a 12-well plate. After 12 h, the cells were fixed with 4% paraformaldehyde (Biosharp, China) for 20 min and then permeabilized with 0.1% Triton X-100 (Macklin, China) for 20 min at room temperature. Subsequently, cells were blocked with 10% bovine serum albumin (BSA) (BioFroxx, Germany) for 1 h at room temperature, followed by overnight incubation with primary antibodies at 4 °C. The samples were then allowed to return to room temperature for 40 min. After being washing with PBS, the cells were incubated with secondary antibody at 1:500 dilution (Boster, China, Cat. No. BA1142) for 1 h at room temperature. Finally, the slides were sealed using an anti-fluorescence quenching agent with DAPI (Abbkine, China, Cat. No. BMU107). The cells were visualized and photographed using a fluorescence microscope (Olympus, Japan).

### Immunohistochemistry staining

The Immunohistochemistry (IHC) staining procedure was conducted following the protocol described previously 25. Formalin-fixed, paraffin-embedded (FFPE) tissues were cut into 4 μm sections. After deparaffinization, the sections were rehydrated and subjected to antigen retrieval by microwaving in 0.01 mol/L sodium citrate buffer (Boster, China, Lot. No. 17F20B24) for 20 min. The sections were then incubated at 4 °C overnight with primary antibodies. Immunostaining was performed using the Immunohistochemistry Kit (Elabscience, China, Cat. No. E-IR-R215) according to the manufacturer's instructions. Subsequently, the slides were counterstained with hematoxylin (Biosharp, China) and mounted in dimethylbenzene. Protein staining was evaluated under a light microscope at 400 × magnification. The staining intensity was scored manually by two independent experienced pathologists as follows: 0 (negative), 1 (weak), 2 (moderate), and 3 (strong). The percentage of positive tumor cells was graded into five categories (percentage scores): 0 (0%), 1 (1-25%), 2 (26-50%), 3 (51-75%), and 4 (76-100%). The overall score of each sample was determined using the formula: overall score = intensity score × percentage score. Total scores of 0-2, 3-6, 7-9, and 10-12 were classified as -, +, ++, and +++, respectively. Tumor cells in five randomly selected fields were analyzed for total scores or integrated optical density (IOD) using ImageJ (Version Fiji, National Institutes of Health, USA). Tissues with score>6 (the median score) were considered as high CHAF1B expression group, and tissues with score ≤ 6 were classified as low CHAF1B expression group. The antibodies used in this study are listed in Table S1.

### Animal xenograft models

5~6-week-old male BALB/c nude mice were purchased from Guangdong Medical Laboratory Animal Center (Guangdong, China) and housed under a 12-hour light/dark cycle, provided with standard laboratory food and water. A total of 20 mice were randomly assigned to two groups: scramble and shCHAF1B, with 10 mice in each group. 5×10^6^ Huh7 cells transfected with either scramble or shCHAF1B were resuspended in 150 μL of DMEM and injected subcutaneously into the right flank of each mouse. Tumor volume was calculated using the formula: 0.5236 L1×(L2)^2^, where L1 represents the long axis and L2 represents the short axis of the tumor 26. Once the tumor volume became palpable (approximately 50 mm³), the mice in both the scramble and shCHAF1B groups were further divided into PBS and sorafenib treatment groups (n = 5/group). Then, the mice received an oral administration of PBS or sorafenib (15mg /kg/day) for 21 days. After 3 weeks of treatment, the mice were sacrificed, and the solid tumors were excised, weighed, and fixed in 4% paraformaldehyde. The study has been approved by the Committee on the Ethics of Animal Experiments of the Fifth Affiliated Hospital of Sun Yat-sen University (NO. 00474) and the whole research process complies with ethical guidelines.

### RNA sequencing and bioinformatics analysis

To explore the underlying mechanism by which CHAF1B contributes to the malignant phenotype of HCC, CHAF1B-overexpressing HepG2 cells and CHAF1B-knockdown HepG2 cells, along with their respective control cells, were collected for RNA isolation followed by RNA sequencing (RNA-seq). Total RNA was extracted from the cultured cells using Trizol reagent (Ambion, USA) according to the manufacturer's instructions. RNA concentration and quality was evaluated with a Nanodrop2000 spectrophotometer (Nanodrop, USA). Then, the electrophoresis on a denaturing agarose gel was performed to detected the RNA integrity and DNA contamination. RNA-seq analysis was conducted by the Cosmos Wisdom Corporation (Hangzhou, China). In brief, the RNA-seq libraries were performed using pretreated RNAs with TruseqTM RNA sample prep Kit (Illumina, USA) following the manufacturer's recommendations. Sequencing was conducted by using an Illumina Novaseq 6000 platform. HISAT2^27^ software was used to count the read numbers mapped to each gene. FPKM (fragments per kilobase of transcript sequence per millions base pairs sequenced) of each gene was calculated based on the length of the gene and the read counts mapped to this gene. Different expressed genes (DEGs) were evaluated by using the DESeq2^28^ R package (4.4.1). The resulting *p* values were adjusted using the Benjamini and Hochberg's approach for controlling the false discovery rate (FDR). Genes with an adjusted *p* value < 0.05 and fold change > 2 were assigned as differentially expressed.

For TCGA and GEO data analysis, RNA-seq data and corresponding clinical data for the liver cancer (LIHC) cohort were downloaded from The Cancer Genome Atlas (TCGA) database (https://tcga-data.nci.nih.gov/tcga/). DEGs in TCGA-LIHC were analyzed using the limma^29^ R package with a criterion of FDR < 0.0005 and fold change > 2. Additionally, RNA-seq data for sorafenib-resistant cells and their corresponding control cells were obtained from the Gene Expression Omnibusdatabase (GEO) database (GEO number: GSE213615) (https://www.ncbi.nlm.nih.gov/geo/). DEGs of HCC-SR cells compared to their parental controls were analyzed using limma with a criterion of FDR < 0.0005 and fold change > 1.

### Statistical analysis

The relationships between CHAF1B expression and relevant patient and tumor characteristics were assessed using the chi-squared test. Overall survival was calculated from the date of HCC resection until the patient were lost to follow-up or died. Recurrence-free survival (RFS) was calculated from the date of the HCC resection to the time of first recurrence. Patients who were lost to follow-up or who died from causes unrelated to HCC were treated as censored events. Kaplan-Meier plots and Cox proportional hazard regression analysis were employed to identify the prognostic factors. A univariate Cox proportional hazards regression analysis was conducted to analyze the associations between the OS and the molecular changes or clinical characteristics. Results are expressed as mean ± standard deviations (SD). Statistical significance was determined using the Student's *t*-test for variables with a normal distribution and the Mann-Whitney *U* test for variables that did not conform to a normal distribution for two sample groups. Statistical significance was determined using the Kruskal Wallis Test for the variables of the three sample groups. For correlation analysis, Pearson's correlation test was used. Data were analyzed using SPSS Statistics 25.0 (IBM, USA), ImageJ (Version Fiji, National Institutes of Health, USA) and GraphPad Prism 8 (Insightful Science, USA). *P* < 0.05 was considered statistically significant.

## Results

### CHAF1B expression may be associated with sorafenib resistance

**Figure 1:**
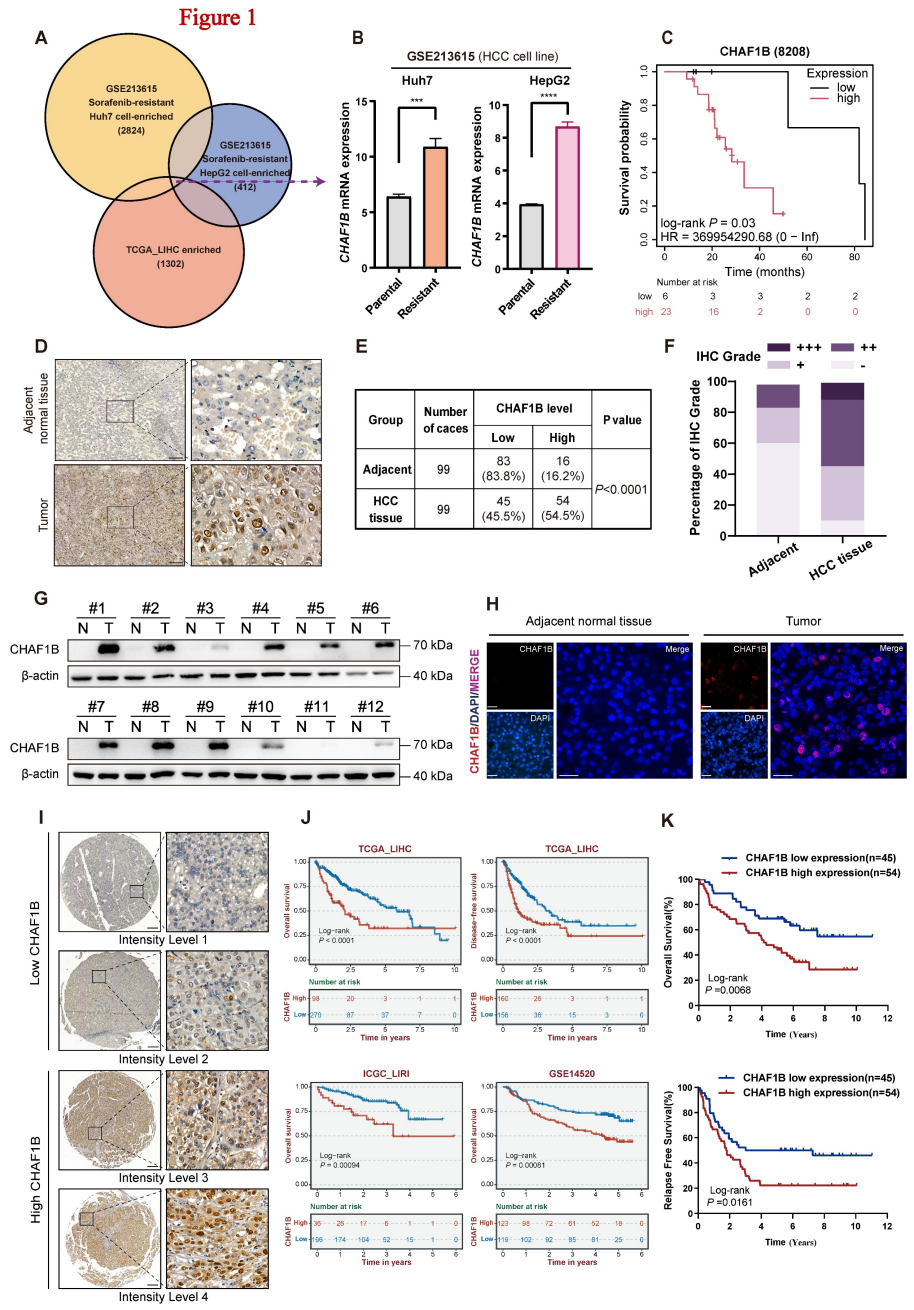
CHAF1B is upregulated in clinical HCC samples and sorafenib-resistant cells, correlating with poor prognosis. (A) Venn diagram showing the intersection of common upregulated differentially expressed genes (DEGs) identified between GEO database (GSE213615) and TCGA database (TCGA-LIHC). (B) CHAF1B RNA expression levels in HCC-SR cells compared to their parental controls from the GSE213615 database. (C) Kaplan-Meier's overall survival analysis for sorafenib-treated patients with HCC according to low or high CHAF1B protein levels (n = 29). (D and I) Representative IHC images showing different levels of CHAF1B expression in 99 HCC tissues and adjacent non-tumor tissues. Scale bars: 200 µm. (E) Chi-square analysis of the CHAF1B levels in 99 HCC tissues and adjacent non-tumor tissues (*P* < 0.0001). (F) The expression level distribution of CHAF1B in HCC tissues and adjacent non-tumor tissues. (G and H) Analysis of the CHAF1B protein levels in HCC clinical samples by western blot and immunofluorescence Scale bars: 50 µm. (J) Kaplan-Meier analysis of the overall survival and disease-free survival using the TCGA-LIHC cohort, ICGC-LIRI cohort and GSE14520 cohort according to CHAF1B expression. (K) The clinical significance of CHAF1B expression in overall survival and relapse-free survival was confirmed in the SYSU-HCC cohort by Kaplan-Meier survival analysis.

To identify the potential candidates involved in sorafenib resistance in HCC, we analyzed the GSE213615 dataset from the GEO database (Table S3 and S4) along with TCGA-LIHC dataset (Table S5). The GSE213615 dataset comprises RNA sequencing data from paired parental and sorafenib-resistant Huh7 as well as HepG2 cell lines 30. Through this analysis, we identified differentially expressed genes (DEGs) demonstrating consistent overexpression across all three transcriptomic datasets as potential molecular correlates of sorafenib resistance (Fig. 1A and Table S6). Among these candidates, CHAF1B exhibited significantly upregulation in sorafenib-resistant HCC cells (Fig. 1B). Clinical correlation analysis using TCGA-LIHC data further demonstrated that elevated CHAF1B expression was significantly associated with reduced survival probability in sorafenib-treated patients (Fig. 1C). Taken together, these findings suggested that CHAF1B may contribute, at least in part, to sorafenib resistance in HCC.

### Increased CHAF1B expression predicts poor clinical outcome in HCC patients

To further investigate the potential role of CHAF1B in the pathogenesis of human HCC, we systematically analyzed its expression patterns and clinical significance. Analysis of TCGA data revealed significant CHAF1B upregulation in HCC tissues compared to normal controls (Fig. S1A, B). Survival analysis across three independent cohorts (TCGA-LIHC, ICGC, and GSE14520) consistently demonstrated that elevated CHAF1B expression was associated with reduced overall and disease-free survival (Fig. 1J). Moreover, TCGA-LIHC data showed a positive correlation between CHAF1B expression and tumor grade progression (Fig. S1C).

Next, we validated these results using our institutional cohort of 99 HCC patients. IHC analysis demonstrated significantly higher CHAF1B expression in tumor tissues compared to adjacent non-tumor tissues (Fig. 1D-F). Western blot and immunofluorescence analyses yielded consistent results (Fig. 1G, H). Based on IHC staining intensity, HCC patients were categorized into low- and high-CHAF1B groups (Fig. 1I). We found that high CHAF1B expression correlated with advanced TNM stages (Table 2) and served as an independent prognostic factor for both decreased overall survival and increased recurrence rates (Fig. 1K and Table S7, 8).

### CHAF1B promotes HCC cell proliferation, migration, invasion and anti-apoptosis in vitro

**Figure 2:**
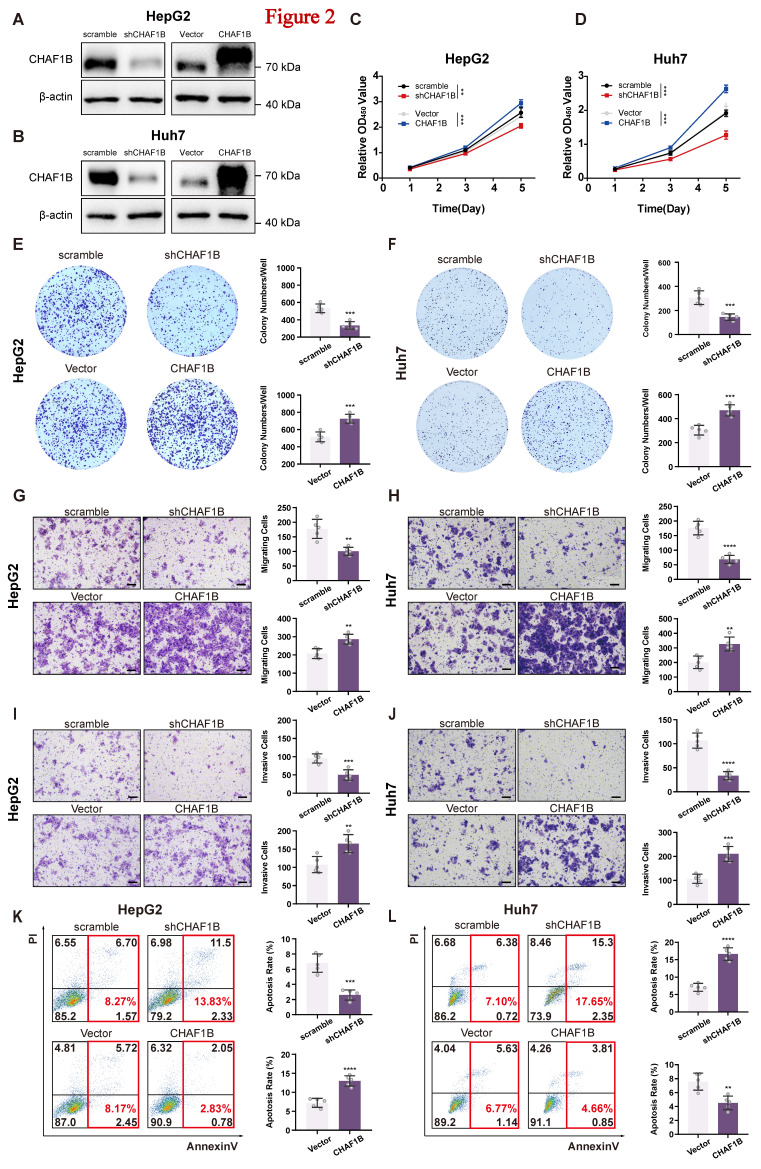
CHAF1B promotes cell proliferation, migration, invasion and anti-apoptosis abilities in HCC cells. (A and B) CHAF1B expression in HCC cells (Huh7 and HepG2) stably transduced with lentivirus was assessed by western blot. (C and D) The effect of CHAF1B on cell proliferation was determined by CCK8 assay. (E and F) Colony formation was performed to validate the impact of CHAF1B on cell proliferation. (G-J) Transwell assays were used to evaluate the effect of CHAF1B on migration and invasion of the indicated cells. Scale bars: 100 µm. (K and L) The flow cytometry was performed in cells with CHAF1B knockdown or overexpression. The percentage of cells in different states was indicated. Data are presented as mean ± SD for five independent experiments, *P < 0.05, **P < 0.01, ***P < 0.001, ****P < 0.0001.

To confirm whether CHAF1B has a tumor-promoting function in HCC, we performed gain- and loss-of-function studies in Huh7 and HepG2 cell lines. Successful modulation of CHAF1B expression was confirmed at both protein (western blot, immunofluorescence) and mRNA (RT-qPCR) levels (Fig. 2A, B and Fig. S2). Functional assays revealed that overexpression of CHAF1B significantly enhanced HCC cell growth in both Huh7 and HepG2 cells (Fig. 2C, D). Consistent results were observed from colony formation assays (Fig. 2E, F). Additionally, CHAF1B overexpression resulted in a marked increase in migration (Fig. 2G, H) and invasion abilities (Fig. 2I, J) of the tumor cells. Furthermore, overexpression of CHAF1B in Huh7 and HepG2 cells was associated with decreased apoptosis (Fig. 2K, L). Conversely, CHAF1B knockdown produced opposing effects, suppressing proliferative, migratory, and invasive capacities while promoting apoptosis (Fig. 2C-L). Together, these consistent findings across multiple functional assays strongly support an oncogenic role for CHAF1B in HCC pathogenesis.

### CHAF1B is negatively correlated with HCC cell response to sorafenib

**Figure 3:**
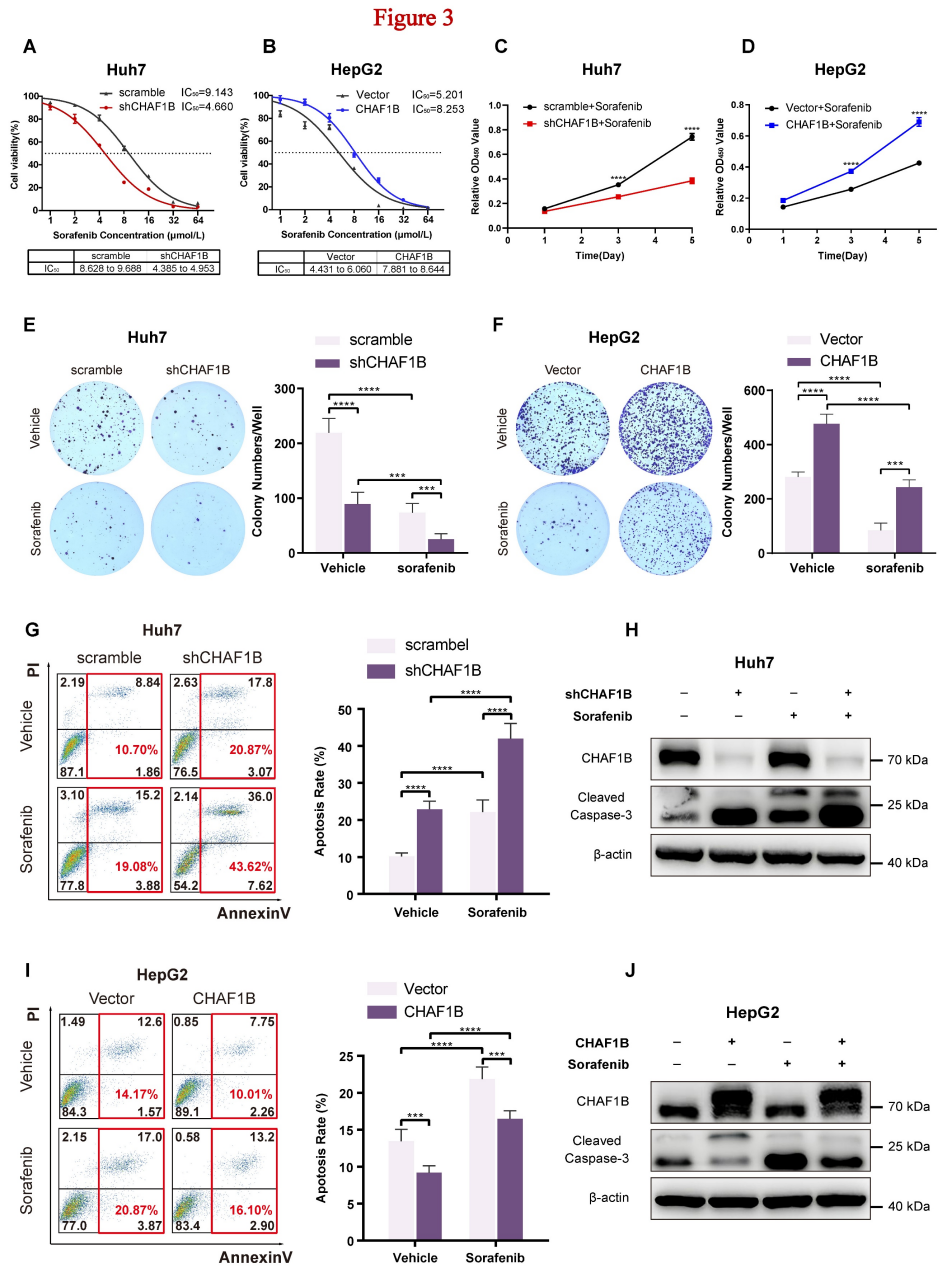
CHAF1B is negatively correlated with the HCC cells' response to sorafenib. (A and B) Cell viability of Huh7 (A) and HepG2 (B) cells upon CHAF1B knockdown or overexpression was analyzed by CCK8 assay. (C and D) CCK8 assay to detect the growth inhibition of sorafenib on Huh7 (C) and HepG2 (D) cells by the condition of down-regulation of CHAF1B or up-regulation of CHAF1B. (E and F) Colony formation was performed to validate the impact of CHAF1B on HCC cells' response to sorafenib. (G and I) CHAF1B-knockdown (G) or CHAF1B-overexpression (I) and the control cells were treated with sorafenib (5 µM) for 24h and were analyzed for apoptotic rate by flow cytometry. (H and J) Expression of apoptotic markers was assessed in Huh7 CHAF1B-knockdown cells (H) and HepG2 CHAF1B-overexpression cells (J) by western blot. Data are presented as mean ± SD for five independent experiments, *P < 0.05, **P < 0.01, ***P < 0.001, ****P < 0.0001.

Because our above results showed significantly higher CHAF1B expression in sorafenib-resistant HCC cell lines, we further investigate the role of CHAF1B in mediating sorafenib resistance in HCC. Compared to the control, IC_50_ values of CHAF1B-knockdown Huh7 cells markedly decreased (9.143 µmol/L *vs.* 4.660 µmol/L) (Fig. 3A), while IC_50_ values of CHAF1B-overexpression HepG2 cells increased (5.201 µmol/L *vs.* 8.253 µmol/L) (Fig. 3B). Furthermore, the proliferation assay demonstrated that sorafenib (4 µmol/L) significantly inhibited the growth of CHAF1B-knockdown Huh7 cells, while having only a minimal effect on the control cells (Fig. 3C). In contrast, sorafenib (4 µmol/L) exhibited only mild inhibition of growth in CHAF1B-overexpression HepG2 cells, whereas it markedly suppressed the proliferation of the control cells (Fig. 3D). Consistent results were observed in colony formation assays (Fig. 3E, F).

As sorafenib reportedly induces apoptosis in HCC cells 4, we examined the impact of CHAF1B alteration on apoptosis. The apoptosis of HCC cells was detected using Annexin V/PI flow cytometry and western blot analysis. The results showed that CHAF1B knockdown induced cell death, evidenced by an increase in apoptotic cells (Fig. 3G) and cleaved forms of caspase-3 (Fig. 3H); while CHAF1B overexpression exhibited the opposite way (Fig. 3I, J). Altogether, our data revealed that CHAF1B is negatively correlated with HCC cell response to sorafenib, and significant antitumor activity could be achieved through the combination of CHAF1B knockdown and sorafenib treatment in HCC.

### Knockdown of CHAF1B confers HCC cells sensitivity to sorafenib in vivo

**Figure 4:**
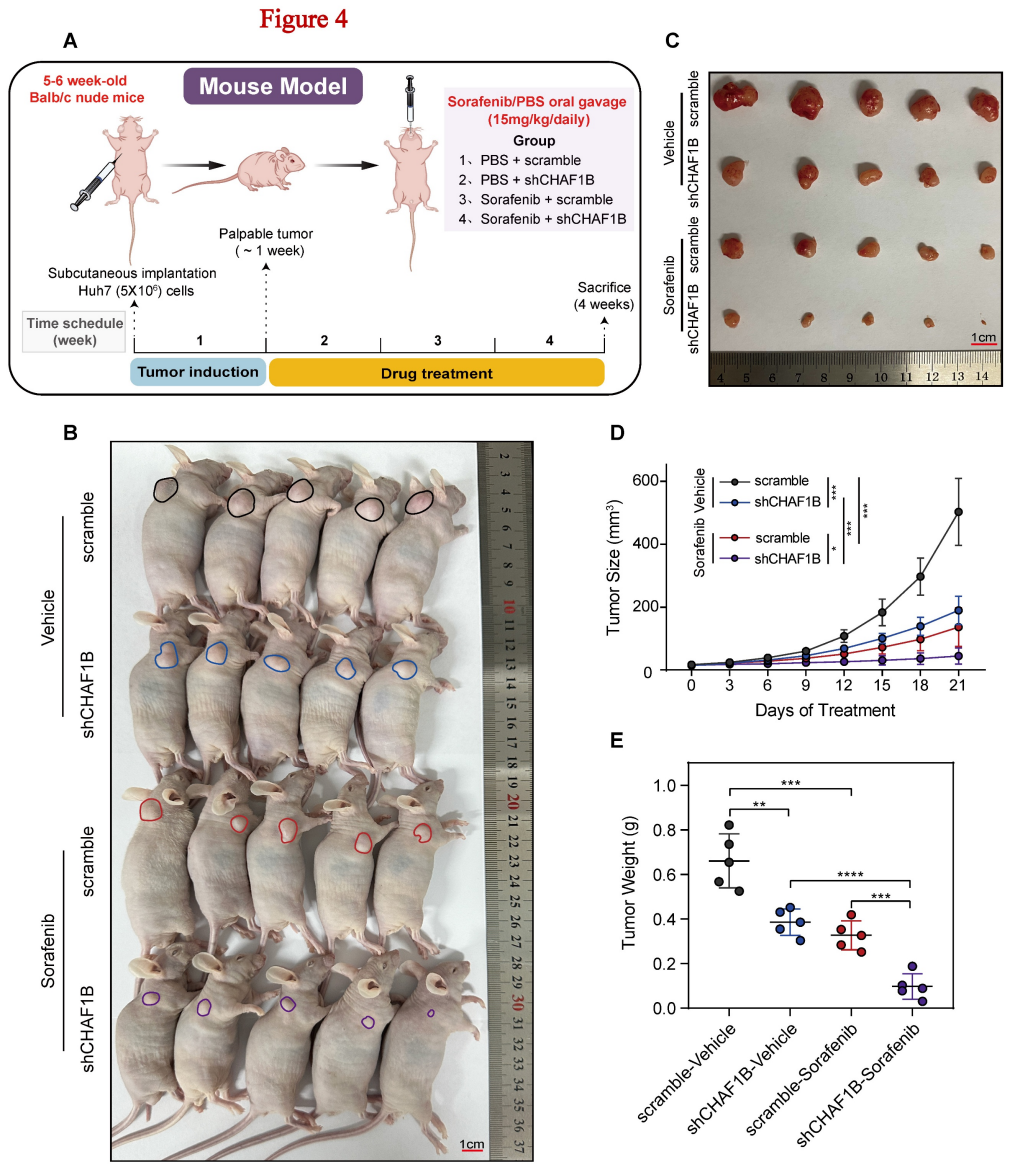
Knowdown of CHAF1B inhibits tumor growth and enhances sensitivity to sorafenib *in vivo*. (A) Schematic image of the Huh7 xenografted model. After tumor establishment (palpable tumor, ~ 1 week), mice were treated with PBS or sorafenib, respectively (n = 5). (B and C) Representative photographs of tumors developed in mice. (D) Tumor volumes were measured every 3 days, and tumor growth curves were created for each group. (E) Weight of dissected tumors from each group. Data is shown as the mean ± SD, *P < 0.05, **P < 0.01, ***P < 0.001, ****P < 0.0001.

To determine whether these *in vitro* findings could be validated *in vivo*, we employed a subcutaneous xenograft model using Huh7 cells stably transfected with shCHAF1B or an empty vector. Upon tumor establishment, the xenografted mice were administered either PBS or sorafenib via gavage for 21 days (Fig. 4A). Photographs of the mice and the dissected subcutaneous tumors, captured on day 21, are shown in (Fig. 4B, C). The combination of sorafenib and shCHAF1B showed a significant synergistic effect on tumor growth rate and mass (Fig. 4D, E). Taken together, these results suggest that the knockdown of CHAF1B enhances the sensitivity of HCC cells to sorafenib *in vivo*.

### PI3K/Akt/HIF-1α signaling axis is modulated by CHAF1B in HCC

**Figure 5:**
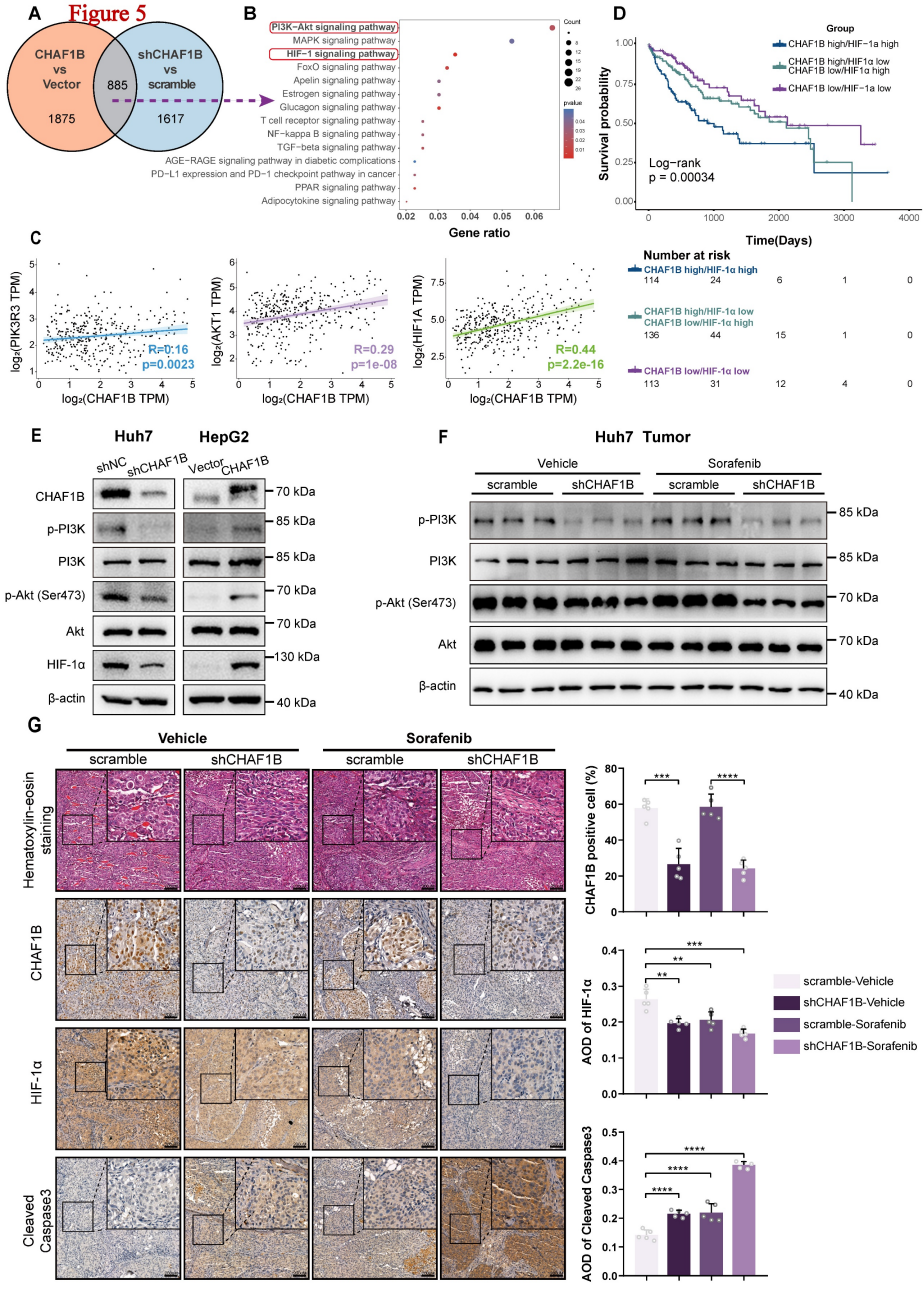
Knowdown of CHAF1B inhibits the PI3K/Akt/HIF-1α signal pathway. (A) Venn diagram showing the intersection of differentially expressed genes (DEGs) in HepG2 cells (OE-CHAF1B/OE-NC, shCHAF1B/sh-NC). (B) KEGG enrichment analysis of DEGs indicates CHAF1B correlates with PI3K/Akt/HIF-1α signal pathway. (C) Pearson correlation analysis of the correlation between CHAF1B expression and relative genes in HCC patients from the TCGA database. (D) Survival analysis of three subgroups (CHAF1B high/HIF-1α high, CHAF1B high/HIF-1α low or CHAF1B low/HIF-1α high, CHAF1B low/HIF-1α low) from the TCGA database. (E) Western blot analysis of markers of PI3K/Akt/HIF-1α signal pathway in Huh7 cells with knockdown of CHAF1B and HepG2 cells with overexpressing CHAF1B. (F) Expression of PI3K/Akt in Huh7 xenograft tumors was detected by western blot. (G) H&E and IHC staining of CHAF1B, HIF-1α and Cleaved-Caspased3 were conducted in serial sections of tumors from each groups. Scale bars: 200 µm. Data represents the mean ± SD of the IHC score of five independent animals for each group. **P < 0.01, ***P < 0.001, ****P < 0.0001.

To elucidate the molecular mechanisms underlying CHAF1B's oncogenic role in HCC, we performed transcriptomic profiling of CHAF1B-manipulated HepG2 cells. RNA sequencing revealed 2,760 differentially expressed genes (DEGs) in CHAF1B-overexpressing cells and 2,502 DEGs in CHAF1B-knockdown cells compared to controls (Fig. S3A-E and Table S9, 10]. Intersection analysis identified 885 common DEGs (Fig. 5A and Table S11), with KEGG pathway enrichment highlighting significant involvement of PI3K/Akt and HIF-1α signaling pathways (Fig. 5B and Table S12). Bioinformatic analysis of TCGA data confirmed strong positive correlations between CHAF1B expression and PI3K, Akt, and HIF-1α mRNA levels in HCC patients (Fig. 5C). Clinically, TCGA-LIHC patients stratified by combined CHAF1B/HIF-1α expression showed markedly different survival outcomes (log-rank p=0.00034), with the CHAF1B high/HIF-1α high subgroup demonstrating the poorest 5-year overall survival (Fig. 5D).

To determine whether CHAF1B could regulate the PI3K/Akt/HIF-1α signaling pathway, we tested the expression of several key members of this pathway in HCC cells using western blot analysis. As predicted, the results showed that overexpression of CHAF1B in HepG2 cells led to increased phosphorylation of PI3K (p-PI3K) and Akt (p-Akt Ser473), as well as HIF-1α expression, while CHAF1B knockdown in Huh7 cells produced reciprocal effects (Fig. 5E). These findings were corroborated in vivo, where CHAF1B-knockdown xenografts showed reduced p-PI3K, p-Akt (Ser473), and HIF-1α levels compared to controls (Fig. 5F, G). Notably, combining CHAF1B knockdown with sorafenib treatment significantly enhanced caspase-3 cleavage.

Collectively, these multi-omics analyses and functional validations demonstrate that CHAF1B promotes HCC progression through activation of the PI3K/Akt/HIF-1α signaling axis.

### Inhibition of PI3K/Akt/HIF-1α activity re-sensitize HCC to CHAF1B-mediated sorafenib resistance

To investigate whether PI3K/Akt/HIF-1α pathway activation is essential for CHAF1B-induced sorafenib resistance, we treated CHAF1B-overexpressing HepG2 cells with sorafenib alone or in combination with pathway-specific inhibitors: PI3K inhibitor BKM120, Akt inhibitor MK2206, or HIF-1α inhibitor LW6.

**Figure 6:**
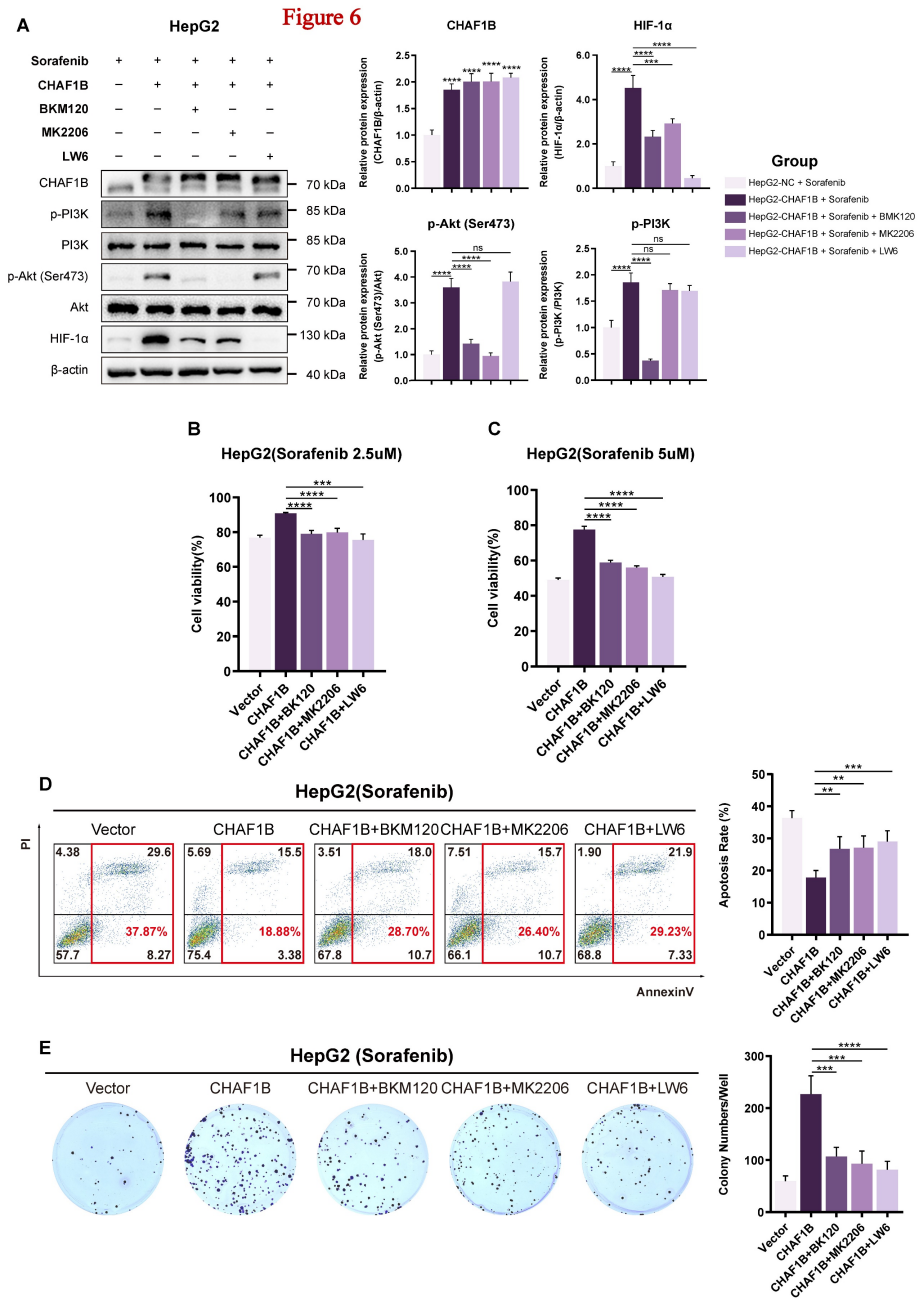
Inhibition of PI3K/Akt/HIF-1α activity disrupts tolerance to sorafenib in CHAF1B-overexpression HepG2 cells. (A) Western blot results depicting the protein expression levels of HIF-1α, PI3K, p-PI3K, AKT, p-Akt (Ser473), CHAF1B in HepG2 cells with/without overexpressing CHAF1B treated with sorafinib and BKM120 or MK2206 or LW6. (BKM120: PI3K inhibitor; MK2206: Akt inhibitor; LW6: HIF-1α inhibitor). (B and C) HepG2 cells were treated with sorafenib alone (2.5 µM or 5 µM) or the combination of sorafenib with BKM120 (0.25 µM), MK2206 (2 µM), or LW6 (12 µM), and cell viability was measured by CCK8 after 48h. (D) The flow cytometry was performed to evaluate the effect of inhibition of PI3K/Akt/HIF-1α activity on CHAF1B-overexpression HepG2 cells treated with sorafenib. (E) Colony formation was performed to evaluate the effect of inhibition of PI3K/Akt/HIF-1α activity on CHAF1B-overexpression HepG2 cells treated with sorafenib. Data are presented as mean ± SD for five independent experiments, *P < 0.05, **P < 0.01, ***P < 0.001, ****P < 0.0001.

To ensure that these three inhibitors effectively inhibited PI3K phosphorylation, Akt phosphorylation and the activation of HIF-1α, respectively, we first treated HepG2 cells with increasing concentrations of BKM120, MK2206, or LW6. The CCK8 assay and western blot analysis indicated that as the concentrations of BKM120, MK2206, or LW6 increased, the inhibitory effects significantly improved (Fig. S4). The appropriate concentrations selected for the following study were 0.25 µM for BKM120, 2 µM for MK2206, and 12 µM for LW6. The western blot results indicated that the activation of p-PI3K, p-Akt and HIF-1α in CHAF1B-overexpression HepG2 cells could be reversed following treatment with BKM120 treatment. In contrast, the activation of p-Akt and HIF-1α was reversed after treatment with MK2206, while only HIF-1α was reversed after treatment with LW6 (Fig. 6A). Meanwhile, treatment with PI3K/Akt/HIF-1α inhibitor mostly reversed the sorafenib tolerance in CHAF1B-overexpressing HepG2 cells, as revealed by the cytotoxicity experiment (Fig. 6B, C). Similar results were also found in the cell apoptotic ratio measured by flow cytometry and the number of colonies measured by colony formation (Fig. 6D, E). Taken together, these findings demonstrate that pharmacological inhibition of the PI3K/Akt/HIF-1α axis reverses CHAF1B-mediated sorafenib resistance, suggesting this pathway as a key mechanistic link between CHAF1B overexpression and treatment failure.

## Discussion

As the first approved first-line systematic treatment for HCC, sorafenib effectively improves the survival of patients with advanced HCC 5, 31. However, its therapeutic efficacy remains substantially limited by primary and acquired resistance, with objective response rates of below 30% 9. Therefore, to enhance the efficacy of sorafenib, it is urgent to investigate the mechanisms of sorafenib resistance and identify potential therapeutic targets. Our study establishes CHAF1B as a key mediator of sorafenib resistance in HCC through comprehensive in vitro and in vivo investigations. we first identified that CHAF1B upregulation in both HCC tissues and sorafenib-resistant cell lines, with elevated expression correlating significantly with poor patient prognosis, particularly in sorafenib-treated cohorts. Mechanistically, we discovered that CHAF1B could activate the PI3K/Akt/HIF-1α signaling pathway, thereby enhancing the intrinsic resistance of HCC cells to sorafenib. Notably, CHAF1B knockdown sensitized HCC cells to sorafenib-induced cytotoxicity and apoptosis, suggesting its potential as a therapeutic target.

CHAF1B, the p60 subunit of chromatin assembly factor-1 (CAF-1), plays essential roles in DNA replication-coupled nucleosome assembly 32. Emerging evidence implicates CHAF1B is highly expressed across diverse malignancies, including leukemia, high-grade gliomas, melanomas, endometrial tumors, prostate cancer, and so forth 12-15, 17, 18, 33, 34. The overexpression of CHAF1B is associated with histological grading and may serve as an indicator of poor prognosis in cancer patients. Furthermore, it has been reported that CHAF1B is linked to tumor progression to tumor progression and invasiveness 12, 21. Consistent with these reports, our TCGA-LIHC analysis and institutional cohort data confirm CHAF1B overexpression in HCC, where it correlates with advanced TNM staging and reduced overall (OS) and relapse-free survival (RFS). Functional assays further demonstrated that CHAF1B promotes aggressive oncogenic phenotypes—enhancing invasion, migration, clonogenicity, and apoptosis resistance—while its inhibition reverses these effects.

CHAF1B has emerged as a promising predictive biomarker for therapeutic response monitoring in cancer 17. Accumulating evidence demonstrates its critical role in treatment resistance across malignancies. While previous studies have established that CHAF1B knockdown promotes apoptosis in proliferating cells 35, recent investigations have specifically implicated CHAF1B in chemo- and radioresistance. For instance, Mu *et al*. Demonstrated that CHAF1B overexpression confers radioresistance in nasopharyngeal carcinoma 20, while Lian *et al*. reported that the expression of CHAF1B was elevated in cisplatin-resistant lung adenocarcinoma cells compared to control cells, and knockdown of CHAF1B significantly increased sensitivity to cisplatin 19. Our bioinformatic analysis extended these findings to HCC, revealing significant CHAF1B upregulation in sorafenib-resistant cells (Fig. 1B). Based on this evidence, we hypothesize that CHAF1B is involved in sorafenib resistance in HCC and aim to explore its underlying mechanisms. In the present study, we found that higher CHAF1B protein levels predicted weaker sorafenib sensitivity in patients with HCC. Furthermore, upregulation of CHAF1B increased the viability, clonal formation and anti-apoptosis ability of HCC cells treated with sorafenib, whereas silencing CHAF1B resulted in enhanced cell viability and clonal formation, along with increased anti-apoptotic capabilities. These results confirm that CHAF1B plays a critical role in inducing sorafenib resistance in HCC, consistent with our initial hypothesis.

Dysregulation of various endogenous signaling pathways has been implicated in the development of sorafenib resistance in HCC 9. Among these, the activation of the intrinsic pro-survival pathway PI3K/Akt and the hypoxia-related signaling pathway has garnered significant attention 36. Peter *et al*. uncovered that the dysregulation of KRAS contributes to sorafenib resistance through the PI3K/Akt pathway 37. Liang *et al*. reported that EF24 significantly reduces the expression of HIF-1α and HIF-1α-dependent genes, and that inhibition of HIF-1α by EF24 effectively overcomes sorafenib resistance in HCC 38. Furthermore, Wu *et al*. found that Akt signaling was involved in mediating the stabilization of HIF-1α, which contributed to sorafenib resistance both *in vivo* and *in vitro* 39. In the present study, with the aim of exploring the underlying mechanisms of sorafenib resistance in patients with HCC, we conducted RNA sequencing to identify significant changes in signaling pathway between CHAF1B-interfered HCC cell lines, HepG2 and their control cell lines. The results demonstrated that the PI3K/Akt and HIF-1α signaling pathways were among the top three pathways exhibiting the most significant changes, further emphasizing their importance in sorafenib resistance in HCC. The overexpression of CHAF1B upregulated the expression of p-PI3K, p-Akt (Ser473) and HIF-1α, while knockdown of CHAF1B demonstrated opposing trends *in vitro* and *in vivo*, suggesting that CHAF1B may induce sorafenib resistance through the PI3K/Akt/HIF-1α signaling pathway. To further confirm the requirement for activation of PI3K/Akt/HIF-1α pathway in CHAF1B-mediated sorafenib resistance, the PI3K-specific inhibitor BKM120, the Akt-specific inhibitor MK2206, and the HIF-1α-specific inhibitor LW6 were used to inhibit the activity of phosphorylation of PI3K and Akt (Ser473), as well as HIF-1α expression respectively. The results showed that inactivation of the PI3K/Akt/HIF-1α pathway through the corresponding inhibitors could indeed reverse CHAF1B-mediated sorafenib resistance in HCC cells. Taken together, these results support a key role for PI3K/Akt/HIF-1α activation in CHAF1B-mediated sorafenib resistance. A limitation of this study is that we have not investigated how CHAF1B specifically regulates the downstream PI3K/Akt/HIF-1α signaling pathway, which warrants further exploration. The relationship between CHAF1B and sorafenib sensitivity may offer a novel approach to overcoming drug resistance in HCC and facilitate the development of effective treatment strategies.

## Conclusion

**Figure 7:**
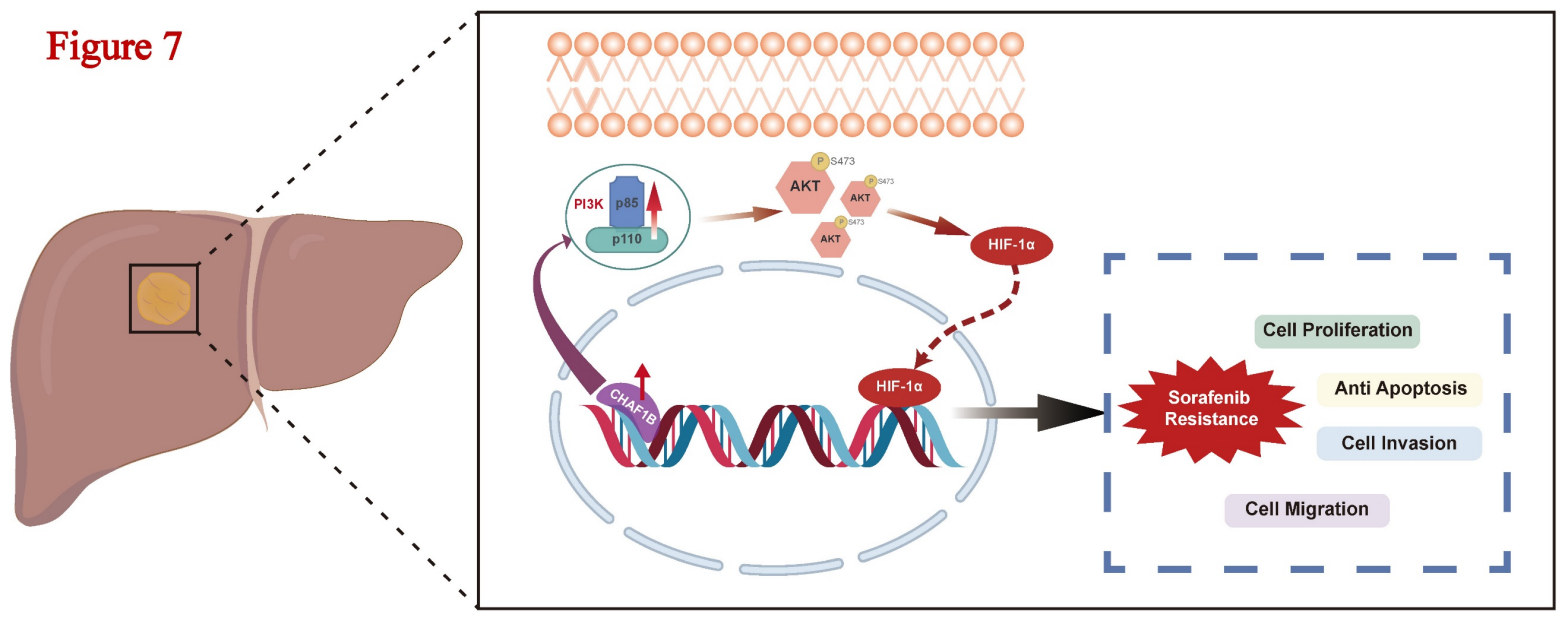
Schematic model of the mechanism underlying CHAF1B on malignant phenotype in HCC. CHAF1B upregulation activates PI3K/Akt signaling through increased phosphorylation of PI3K and Akt (Ser473), leading to upregulation of HIF-1α. This CHAF1B-PI3K/Akt/HIF-1α signaling axis promotes multiple malignant phenotypes including sorafenib resistance, enhanced proliferation, migration, invasion, and apoptosis evasion in HCC cells.

In the current study, we demonstrated that CHAF1B is significantly upregulated in HCC and correlated with poor clinical prognosis. CHAF1B enhances the proliferation, migration, and invasion of HCC cells and contributes to sorafenib resistance through activation of PI3K/Akt/HIF-1α signaling pathway (Fig. 7). These findings suggest that CHAF1B may serve as a potential biomarker and therapeutic target for poor prognostic and for overcoming sorafenib resistance in HCC.

## Supplementary Material

Supplementary figures.

Supplementary tables.

## Figures and Tables

**Table 1:** Primer sequences

Primer name	Primer sequences
F-β-actin	5'-CATGTACGTTGCTATCCAGGC-3'
R-β-actin	5'-CTCCTTAATGTCACGCACGAT-3'
F-CHAF1B	5'-GCGTGGACACCAATGTCAG-3'
R-CHAF1B	5'-GCTCCGGCTCCTTGTTATCAT-3'

**Table 2:** Analysis of correlation between CHAF1B expression and patients' clinicopathological characteristics

Characteristics	Total (%)	CHAF1B expression		*p* value
		High	Low	
Age (years)				
≥55	63(63.6)	33	30	0.567
<55	36(36.4)	21	15	
Gender				
Male	82(82.8)	47	35	0.224
Female	17(17.2)	7	10	
Hepatitis B				
Yes	83(83.8)	43	40	0,213
No	16(16.2)	11	5	
Cirrhosis				
Yes	54(54.5)	28	26	
No	45(45.4)	26	19	0.555
AFP (ng/mL)				
≥400	39(39.4)	26	13	0.051
<400	60(60.6)	28	32	
ALT (U/L)				
≥50	28(28.3)	19	9	0.095
<50	71(71.7)	35	36	
AST (U/L)				
≥40	44(44.4)	25	19	0.685
<40	55(55.6)	29	26	
GGT (U/L)				
≥50	56(56.6)	31	25	0.853
<50	43(43.4)	23	20	
Albumin (g/L)				
≥35	79(79.8)	43	36	0.964
<35	20(20.2)	11	9	
T-Bil (μmol/L)				
≥17.1	30(30.3)	20	10	0.11
<17.1	69(69.7)	34	35	
Number of lesions				
More than one	29(29.3)	20	9	0.064
Only one	70(70.7)	34	36	
Tumor sizes (cm)				
≥5	36(36.4)	24	12	0.067
<5	63(63.6)	30	33	
Pathologic differentiation				
Low-moderate differentiated	75(75.8)	38	37	0.171
High differentiated	24(24.2)	16	8	
Location				
Right lobe	63(63.6)	31	32	0.158
Not right lobe	36(36.4)	23	13	
Completion of capsula				
Yes	72(72.7)	38	34	0.564
No	27(27.3)	16	11	
TNM stage				
Ⅰ~Ⅱ	78(78.8)	38	40	0.025*
Ⅲ~Ⅳ	21(21.2)	16	5	
MVI				
Yes	23(23.2)	15	8	0.241
No	76(76.8)	39	37	

Data were analyzed by chi-squared test or Fisher's exact test AFP, alpha fetoprotein; ALT, alanine aminotransferase; AST, aspartate aminotransferase; GGT, γ-glutamyl transpeptidase; T-Bil, total bilirubin; MVI, microvascular invasion. *p* value with * indicates a statistically significant difference was observed.

## Abbreviations

HCC: Hepatocellular carcinoma
CHAF1B: Chromatin assembly factor 1B
CAF-1: Chromatin Assembly Factor-1
mTKI: Multi-tyrosine kinase inhibitor
FDA: Food and drug administration
OS: Overall survival
STR: Short tandem repeat
DMEM: Dulbecco's Modified Eagle Medium
FBS: Fetal bovine serum
CCK8: Cell counting kit-8
RT-qPCR: Real-Time Quantitative PCR
TCGA: The Cancer Genome Atlas
GEO: Gene Expression Omnibus database
DEGs: Different expressed genes
FDR: False discovery rate
RFS: Recurrence-free survival
SD: Standard deviations
TCGA-LIHC: TCGA liver dataset
