# Supplementary material for: CHAF1B: the hidden culprit behind sorafenib resistance in hepatocellular carcinoma
Source: Int J Med Sci. 2025 Sep 22;22(15):4085–101. doi: 10.7150/ijms.118471 (PMC12492380; doi:10.7150/ijms.118471)
Supplement: Supplementary file 1 — Supplementary figures. [file ijmsv22p4085s1.pdf]

**Supplementary figure legends**

**Figure S1.** CHAF1B expression is elevated in HCC and associated with poor clinical outcomes. (A) HCC datasets from the TCGA dataset showed an increase of CHAF1B mRNA in HCC samples. (B) The mRNA levels of CHAF1B in 44 pairs of HCC and adjacent non-tumor tissues from the TCGA dataset. (C) Subgroup analysis of the TCGA-LIHC cohort exhibited higher mRNA expression levels of CHAF1B in HCC patients with worse histological differentiation grades.

**Figure S2.** Confirmation of stable CHAF1B-overexpression or CHAF1B-knockdown HCC cell lines. (A and B) CHAF1B expression in HCC cells (Huh7 and HepG2) stably transduced with lentivirus was assessed by RT-qPCR. (C) Immunofluorescence confirmed the efficiencies of CHAF1B knockdown in Huh7 cells. (D) Immunofluorescence confirmed the efficiencies of CHAF1B overexpression in Huh7 cells. (E) Immunofluorescence confirmed the efficiencies of CHAF1B knockdown in HepG2 cells. (F) Immunofluorescence confirmed the efficiencies of CHAF1B overexpression in HepG2 cells.

**Figure S3.** Transcriptome sequencing result of HepG2 stable CHAF1B-overexpression or CHAF1B-knockdown cell lines. (A) Bar plot of DEGs in HepG2 cell lines (OE-CHAF1B *vs.* OE-NC, shCHAF1B *vs.* sh-NC). (B) Principal component analysis of HepG2 cells with/without knockdown of CHAF1B. (C) Volcano plot of DEGs in HepG2 cells with/without knockdown of CHAF1B. (D)

Principal component analysis of HepG2 cells with/without overexpressing CHAF1B. (E) Volcano plot of DEGs in HepG2 cells with/without overexpressing CHAF1B.

**Figure S4.** Confirmation of efficiencies of PI3K/Akt/HIF-1α specific inhibitors. (A, C and E) Dose-dependent effects of BKM120 (A), MK2206 (C), or LW6(E) on the viability of HepG2. (B) Western blot analysis shows the effect of PI3K inhibitor BKM120 on the levels of p-PI3K proteins. (D) Western blot analysis shows the effect of Akt inhibitor MK2206 on the levels of p-Akt (Ser473) proteins. (F) Western blot analysis shows the effect of HIF-1α inhibitor LW6 on the levels of HIF-1α proteins.

# Figure S1

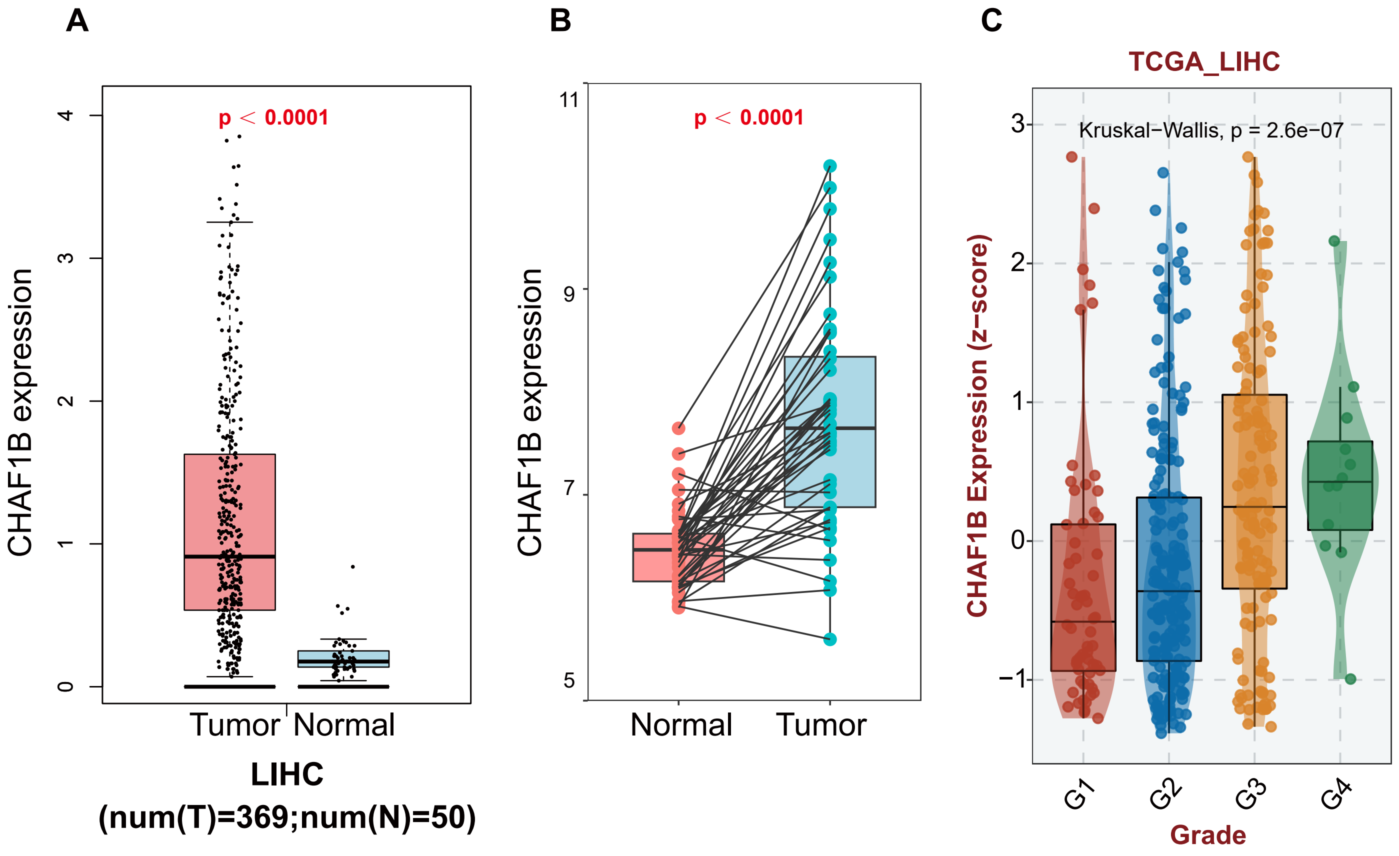

A
B
C
p < 0.0001
CHAF1B expression
Tumor
Normal
LIHC
(num(T)=369;num(N)=50)
p < 0.0001
CHAF1B expression
Normal
Tumor
TCGA_LIHC
Kruskal−Wallis, p = 2.6e−07
CHAF1B Expression (z−score)
G1
G2
G3
G4
Grade

# Figure S2

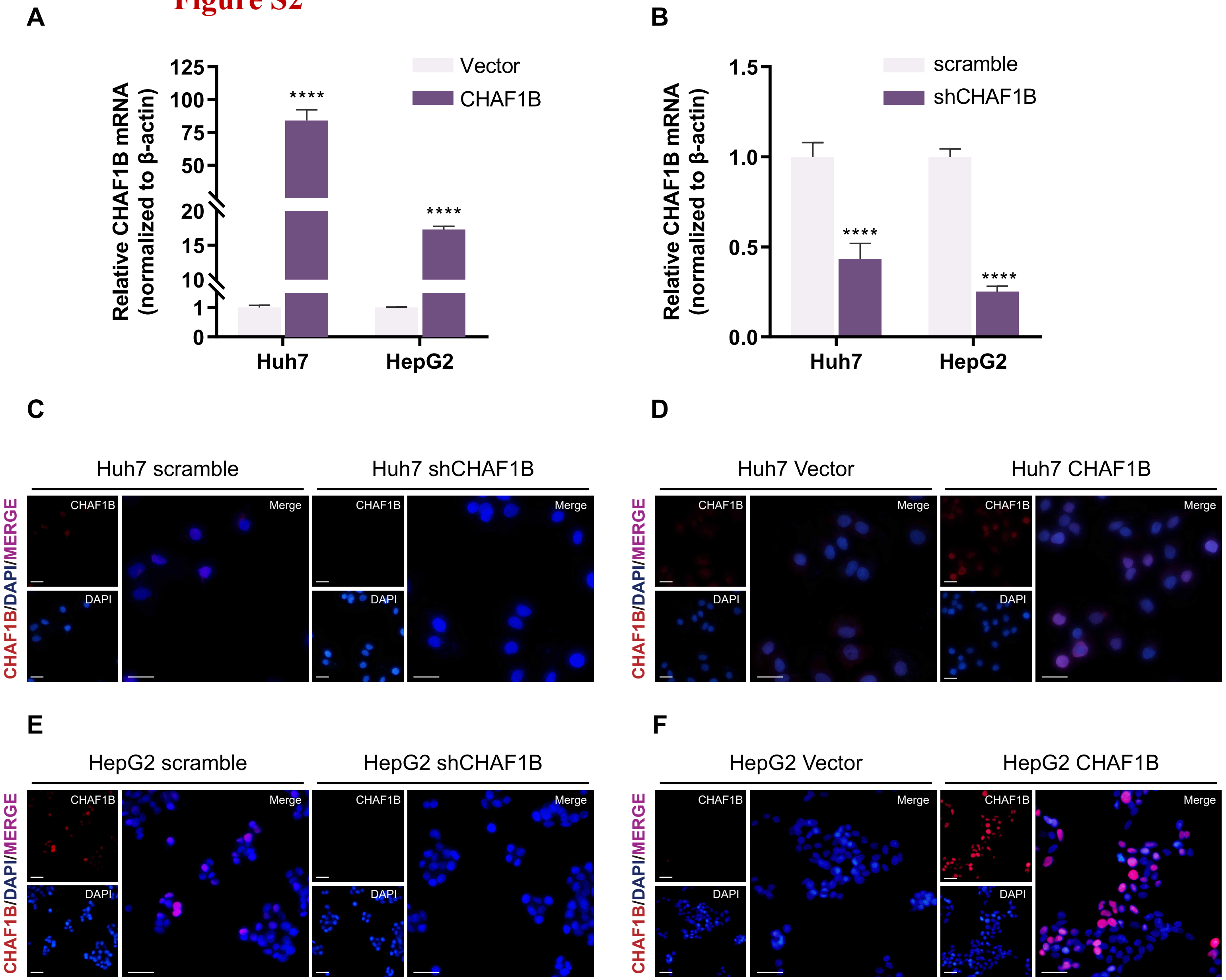
A
B
C
D
E
F
125
100
75
50
20
15
10
1
0
Relative CHAF1B mRNA
(normalized to β-actin)
Vector
CHAF1B
****
****
Huh7
HepG2
1.5
1.0
0.5
0.0
scramble
shCHAF1B
****
****
Huh7 scramble
Huh7 shCHAF1B
Huh7 Vector
Huh7 CHAF1B
HepG2 scramble
HepG2 shCHAF1B
HepG2 Vector
HepG2 CHAF1B
CHAF1B/DAPI/MERGE
CHAF1B
DAPI
Merge

# Figure S3

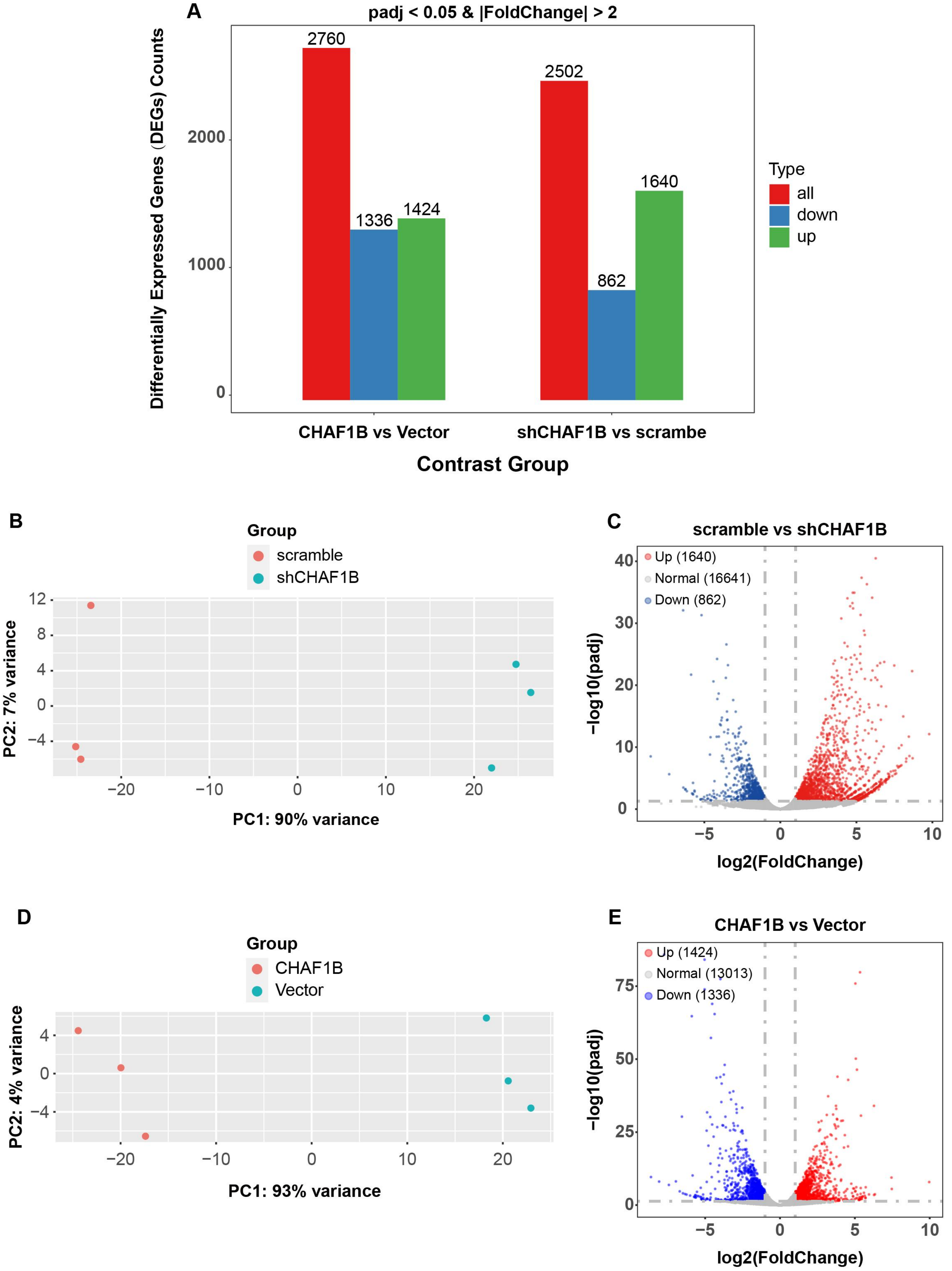
A
padj < 0.05 & |FoldChange| > 2
Differentially Expressed Genes (DEGs) Counts
2760
1336
1424
2502
862
1640
2000
1000
0
CHAF1B vs Vector
shCHAF1B vs scrambe
Contrast Group
Type
all
down
up
B
Group
scramble
shCHAF1B
PC2: 7% variance
PC1: 90% variance
C
scramble vs shCHAF1B
Up (1640)
Normal (16641)
Down (862)
−log10(padj)
log2(FoldChange)
D
Group
CHAF1B
Vector
PC2: 4% variance
PC1: 93% variance
E
CHAF1B vs Vector
Up (1424)
Normal (13013)
Down (1336)
−log10(padj)
log2(FoldChange)

# Figure S4

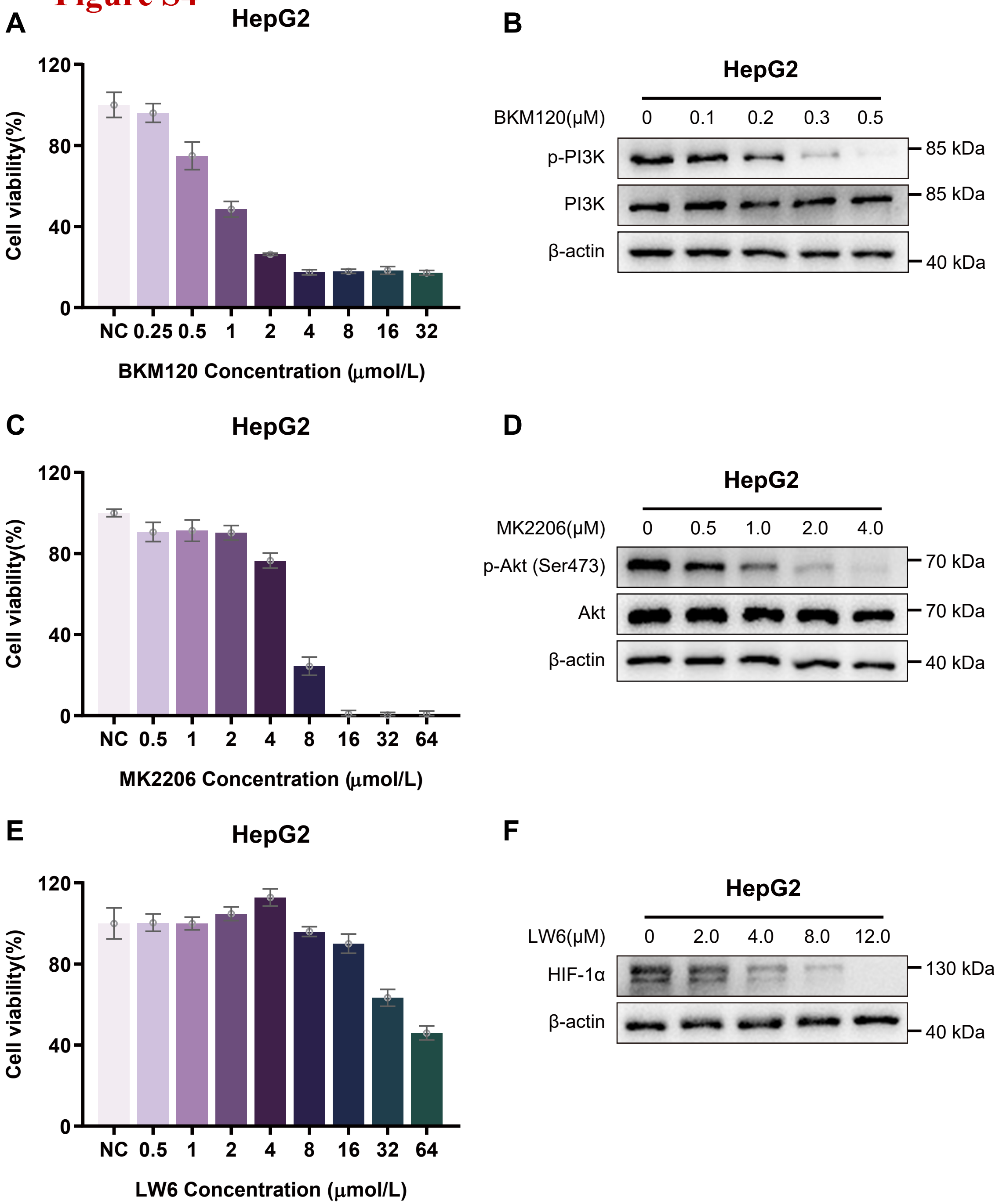
A
HepG2
Cell viability(%)
120
80
40
0
NC 0.25 0.5 1 2 4 8 16 32
BKM120 Concentration (μmol/L)
B
HepG2
BKM120(μM) 0 0.1 0.2 0.3 0.5
p-PI3K
PI3K
β-actin
85 kDa
85 kDa
40 kDa
C
HepG2
Cell viability(%)
NC 0.5 1 2 4 8 16 32 64
MK2206 Concentration (μmol/L)
D
HepG2
MK2206(μM) 0 0.5 1.0 2.0 4.0
p-Akt (Ser473)
Akt
β-actin
70 kDa
70 kDa
40 kDa
E
HepG2
Cell viability(%)
NC 0.5 1 2 4 8 16 32 64
LW6 Concentration (μmol/L)
F
HepG2
LW6(μM) 0 2.0 4.0 8.0 12.0
HIF-1α
β-actin
130 kDa
40 kDa
